# Pediatric Radiation Oncology in the Era of COVID‐19: A Single Institution Analysis

**DOI:** 10.1002/cnr2.70277

**Published:** 2025-07-07

**Authors:** Melisa Pasli, Michael C. Larkins, George Edwards, Megan Goins, Dayana Gonzalez, Cathleen Cook, Andrew W. Ju, Aidan Burke

**Affiliations:** ^1^ East Carolina University (ECU) Brody School of Medicine (BSOM) Greenville North Carolina USA; ^2^ Department of Emergency Medicine Boonshoft School of Medicine at Wright State University Fairborn Ohio USA; ^3^ ECU Department of Pediatrics, Division of Pediatric Hematology/Oncology Greenville North Carolina USA; ^4^ ECU Department of Radiation Oncology Greenville North Carolina USA

**Keywords:** barriers to care delivery, COVID‐19, pediatric oncology, radiation therapy, radiotherapy

## Abstract

**Aims:**

As a result of the COVID‐19 pandemic, health inequities have garnered heightened attention in the public consciousness. In particular, rural access to diagnosis and radiotherapy (RT) for pediatric oncology patients was markedly affected during this period. The fractionated nature of RT creates a transportation burden for this population. We reviewed our institutional experience with pediatric oncologic therapy at a tertiary academic center serving a primarily rural population over a large geographic area.

**Methods:**

Pediatric patients aged ≤ 18 years diagnosed with cancer between 2018 and 2022 at our institution were investigated, and we identified the subset of patients who received RT at our institution. Patients were categorized as pre‐COVID onset (diagnosed between 2018 and January 31, 2020) or post‐COVID onset (diagnosed on or after January 31, 2020, to December 1st, 2022). Chi‐Square and Student's t‐tests were used to elucidate associations between patient demographics and treatment modalities in the pre‐ and post‐COVID onset groups.

**Results:**

A total of 114 patients were identified. For patients that received RT (*n* = 22), 4.5 times more patients traveled from rural counties post‐COVID onset (*p* = 0.027). These patients also saw increased rates of central nervous system (CNS) and non‐hematologic cancer diagnosis (*p* = 0.013 and 0.049, respectively). No difference was seen concerning race, patient age, or average distance traveled (*p* = 0.371, 0.249, and 0.420, respectively). No difference was seen in the estimated transportation cost incurred as a result of RT treatment (*p* = 0.144) or in treatment with concurrent chemotherapy (*p* = 0.245). For the entire cohort, no associations were seen concerning age, race, rural versus urban home county, cancer primary site, or in the prevalence of hematologic‐ or CNS‐based cancers.

**Conclusion:**

Our results highlight the importance of understanding barriers to care to improve outcomes in rural pediatric patients, as the burden of RT may be greater for these patients than for those living in urban counties. Further investigation into barriers to treatment among rural pediatric patients undergoing RT is warranted.

## 1 Introduction

Access to healthcare in the pediatric cancer population was significantly affected during the COVID‐19 pandemic, creating a barrier for access to oncologic treatment. The pandemic was declared a public health emergency on January 31st, 2020, and persisted until May 11th, 2023 [1, 2]. Delays in both diagnosis and treatment were noted to be significant during this period [3]. In particular, rural access to cancer diagnosis and treatment was markedly affected [4]. COVID‐19 has been described as a “syndemic” rather than a “pandemic”, referencing the synergistic interaction of medical conditions, economic, and biological aspects of care [5]. The effect of this interplay on the healthcare system has led to disproportionate downstream effects on certain patient populations [6]. The pediatric population is particularly vulnerable to such effects, and pediatric cancer patients may be more impacted given that pediatric oncology treatment is reliant on a multidisciplinary subspecialty team and multimodal therapy. Prompt evaluation and diagnosis are key for improved outcomes [7]. For example, delays in treatment for pediatric patients with hematologic malignancies during this period were linked to significantly increased morbidity in this population [8]. Therefore, understanding key factors influencing outcomes for these patients is of the utmost importance.

Access to radiation therapy for pediatric cancer patients is a prime example of the healthcare disparity presented by the COVID‐19 pandemic. The fractionated nature of radiotherapy (RT) creates a major transportation burden for pediatric oncology patients who often require up to 6 weeks of once‐daily treatment. Travel restrictions and increased travel costs during the pandemic likely exacerbated this already present issue. During the pandemic, fewer cancer patients traveled extended distances to reach treatment facilities [9, 10]. Although access to telemedicine increased during this time, telemedicine is thought to be inadequate for the initial evaluation of pediatric cancer patients, whose symptoms are often nonspecific [11]. Treatment for cancer also requires many visits for laboratory investigations, procedures, imaging studies, chemotherapy, and RT that cannot be done via telehealth.

The primary aims of this study were two‐fold: to describe the demographics and clinical characteristics of the pediatric cancer patients receiving any type of oncological treatment at our institution and to investigate the distance traveled by pediatric cancer patients and related costs of receiving RT at our institution as potential barriers to care. Since our facility is the only provider of radiation therapy for patients over a large geographic area, namely rural Eastern North Carolina, traveling to radiation oncology treatment creates a significant barrier for this population. As it is unclear in the literature if the COVID‐19 pandemic exacerbated these barriers for this vulnerable patient population, we sought to further evaluate these barriers before and during the pandemic at a tertiary academic center serving a primarily rural population over a large geographic area. A secondary aim of our study was to further heighten the awareness surrounding social determinants of health (SDOH) that were facilitated by the COVID‐19 pandemic. Prior to the pandemic, several studies have demonstrated how healthcare providers tend to attribute social inequalities to a patient's individual qualities rather than their social circumstances [12, 13], though there seems to be some improvement in this awareness over the past several decades [14]. However, the pandemic may have played a part in further increasing awareness around SDOH, evidenced by a 2021 study surveying ICU nurses, which showed they were highly attuned to their patients' social situations [15]. Increased media coverage on SDOH during this time is thought to be a potential mechanism of increasing awareness around SDOH, and we hope our study can do the same through the scientific literature.

## 2 Methods

### 2.1 Overview of Cohort

Pediatric patients aged ≤ 18 years of age diagnosed with cancer between 2018 and 2022 at our institution were identified via chart review after institutional review board (IRB) review; this project was certified exempt (University and Medical Center IRB 22–002292 “Pediatric Oncology Outcomes Database”). Patients were separated into two groups: “pre‐COVID onset” for those diagnosed before January 31, 2020, and “post‐COVID onset” for those diagnosed on or after this date. This date was used as it marks the beginning of the national public health emergency declared as a result of the COVID‐19 pandemic. Our institution remained the only available center for treating the pediatric oncology patients in the cohort throughout the entire timeframe. Of note, records regarding relapse or progression of disease were not available for the patients in the cohort.

### 2.2 Cohort Characterization

**FIGURE 1:**
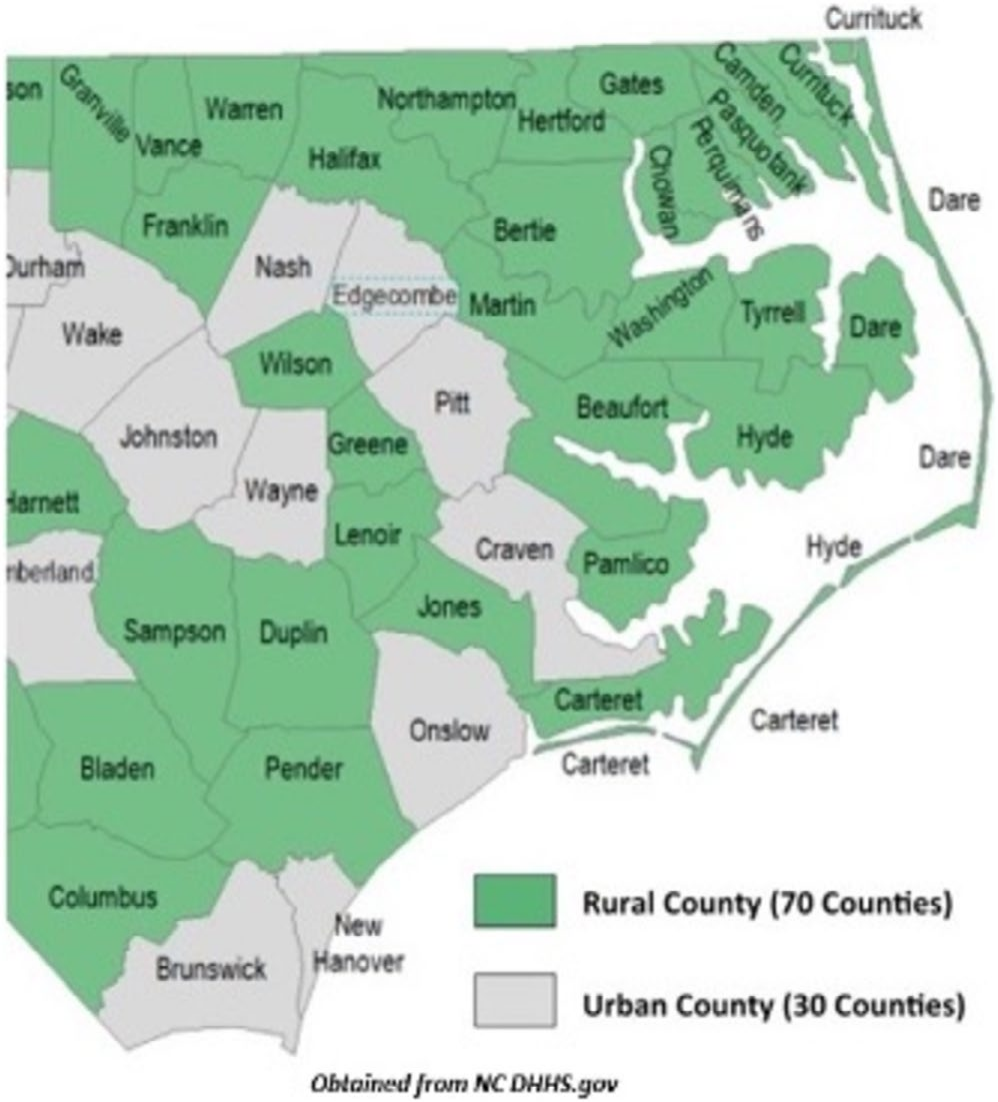
Map depicting rural versus urban home counties near our institution.

Patient cancer diagnosis was performed using criteria from the International Classification of Childhood Cancer, 3rd edition [16]. Patient primary diagnoses were grouped into 11 categories, as depicted in Table 1. Additionally, all patients were stratified into hematologic versus non‐hematologic cancer types, where hematologic cancers were considered leukemias and lymphomas for this analysis. All patients were also stratified as living in rural versus urban counties. Definitions for rural versus urban counties were obtained from state and federal governing bodies for North Carolina, South Carolina, and Virginia, reflecting the home states for our patient population [17, 18, 19]. A map showing rural versus urban counties in NC can be seen in Figure 1.

**TABLE 1:** Pediatric Cancer Categories: Table depicting the cancers included in each category or primary cancer site for pediatric patients diagnosed with cancer at our institution between 2018 and 2022.

Category	Diagnoses include (but not limited to)
Leukemia	ALL, AML, Langerhans histiocytosis
CNS	Hemangioblastomas, Astrocytomas, Glioblastomas
Lymphoma	B‐cell, Hodgkin, non‐Hodgkin
Soft Tissue	Fibromyxoid sarcoma, Leiomyosarcoma
Genitourinary	Renal medullary carcinoma, Choriocarcinoma
Neuroblastoma/PNST	Neuroblastoma, PNST
H&N	Papillary thyroid carcinoma
Bone	Ewing sarcoma, Osteosarcoma
Hepatic	Hepatoblastoma, Hepatocellular carcinoma
GI	Neuroendocrine neoplasms of the GI tract
Thoracic	Pleuropulmonary blastoma

Abbreviations: CNS, Central Nervous System; GI, Gastrointestinal; H&N, Head and Neck; PNST, Peripheral Nerve Sheath Tumor.

Patients that did not undergo surgical treatment for a cancer diagnosis at our institution or for which no records from an outside institution for treatment could be found were collectively placed into a category labelled “No/Unknown/Not Applicable”; this includes patients with diagnoses for which surgery is not considered as standard of care. Patients with an unknown surgical status were included in this category because the nuances of their individualized discussions regarding surgery were not available in our records. For the patients who received RT, variables of interest included travel metrics (distance and estimated time of travel) and cost analyses. Distance was calculated in miles from the patient's primary address to our institution using Google Maps (Google LLC. Released 2023. Version 6.97.0.36020. Mountain View, CA). Cost of travel was calculated for each patient using the IRS‐assessed, national standard mileage rate for operating expenses of travel for medical needs; this rate was determined by the year in which RT was administered. For patients that had RT over two different tax years, the rate used to determine total cost was calculated proportionally with the amount of time RT was conducted over each year (e.g., for a patient that had 50% of their RT in 1 year and 50% in another, half of the total cost was calculated using 1 year's IRS standard rate and the other half was calculated using the other year's rate). The IRS standard tax rates for the years of this study (2018–2022) were pulled from the circulated IRS Form 502 for each corresponding year [20, 21, 22, 23, 24]. The number of fractions was taken from the initial course of radiation treatment to avoid lead time bias. Travel cost was multiplied by the number of fractions and IRS mileage rate, then doubled to account for travel to and from our institution. All patients and RT patients were plotted on pre‐ and post‐COVID onset heat maps based on their primary address using the “US Geographic Heat Map Generator Excel Template” available from Someka (Someka LLC. v6. Izmir, Bayrakli, Türkiye).

### 2.3 Statistical Analysis

Pearson Chi‐Square testing was performed for categorical variables stratified by pre‐ and post‐COVID onset status. In the case of > 20% of expected cell counts having a count < 5, the likelihood ratio (LR) was used instead. For comparison of continuous variables stratified by pre‐ and post‐COVID onset status, two‐tailed Student's *t*‐test was used for analysis. Statistical analysis was performed via SPSS (IBM Corp. Released 2023. IBM SPSS Statistics for Windows, Version 30.0. Armonk, NY: IBM Corp.). A *p*‐value of ≤ 0.05 was used as the threshold for statistical significance, and a 95% confidence interval was utilized for all tests.

## 3 Results

### 3.1 Cohort Characterization and Demographic Analysis

**FIGURE 2:**
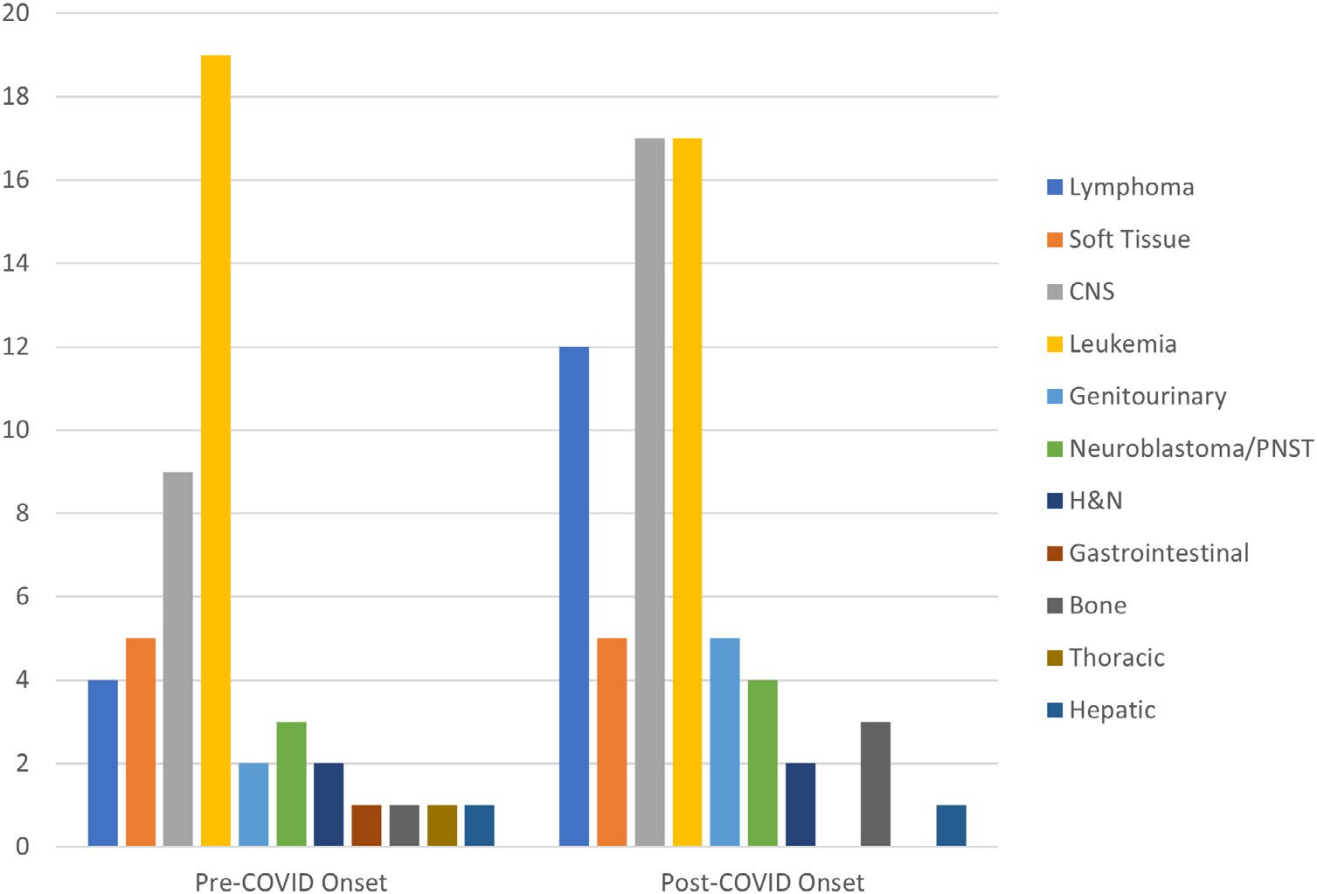
Distribution of cancer type by primary tumor site for all patients (*n* = 114) comparing pre‐ versus post‐COVID onset timeframes. Leukemia, CNS, and lymphoma cancer types were the most common, second‐most common, and third‐most common cancers, respectively.

**FIGURE 3:**
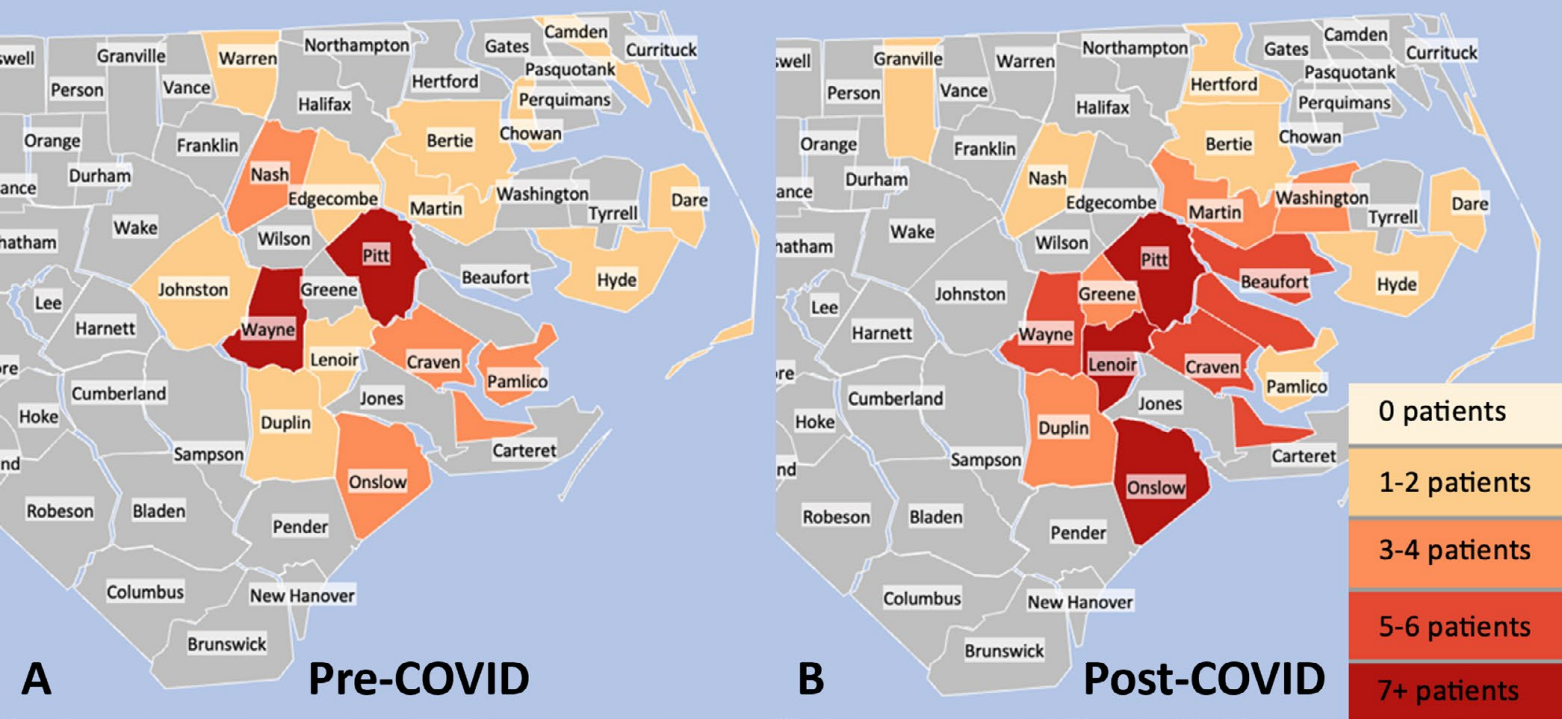
Heat maps of pediatric patients diagnosed with cancer at our institution pre‐ and post‐COVID onset (Panels A and B, respectively). Increasingly dark red corresponds to increasing frequency of patients diagnosed with cancer coming from the labelled county. Pre‐COVID group had 46 patients, post‐COVID group had 68 patients.

A total of 132 pediatric patients were diagnosed with cancer at our institution between 2018 and 2022, shown by year in Table 3. Of these patients, 114 patients were included with complete data regarding race, urban versus rural residence, administration of chemotherapy, and administration of radiation therapy. As noted above in Section 2.2 of the methods, surgical intervention status was not always known. A breakdown of demographics between pre‐ and post‐COVID cohorts can be found in Table 2. The average patient age pre‐COVID onset was 8.7 years (standard deviation (SD) = 6.5 years) and 9.6 years in the post‐COVID group (SD = 5.7 years); no difference in age was noted (*p* = 0.421). The breakdown of cancer types for all patients pre‐ and post‐COVID is shown in Figure 2. Hematologic cancers were the most prevalent (consisting of 16 patients with lymphoma and 36 with leukemia) followed by central nervous system (CNS) tumors (consisting of 26 patients) from both the pre‐ and post‐COVID onset timeframes. Leukemia was the most common pediatric cancer in both pre‐ and post‐COVID onset groups at 19 and 17 diagnoses, respectively. The total number of cancer diagnoses increased in the post‐COVID era in our study cohort, with 48 diagnosed pediatric cancers pre‐COVID onset versus 66 post‐COVID onset. Fifty‐five patients came from counties classified as rural (48%); a heat map of patients diagnosed pre‐ and post‐COVID onset depicting patient home counties can be seen in Figure 3. The average one‐way distance traveled from patient residence to the ECU Health Medical Center for all patients was 47.9 miles (median = 42 miles).

**TABLE 2:** Pediatric patient cohort characterization: demographic and treatment characteristics for pediatric patients diagnosed with cancer at our institution between 2018 and 2022.

	Pre‐COVID onset (%) (*n* = 48)	Post‐COVID onset (%) (*n* = 66)	Total (%) (*n* = 114)
Race			
White	20 (42%)	29 (44%)	49 (43%)
Black	19 (40%)	25 (38%)	44 (39%)
Non‐White Hispanic	0	1 (2%)	1 (1%)
American Indian or Alaska Native	0	1 (2%)	1 (1%)
Mixed	2 (4%)	2 (3%)	4 (4%)
Other	6 (13%)	8 (12%)	14 (12%)
Unknown	1 (2%)	0	1 (1%)
Urban vs. Rural home county			
Urban	28 (58%)	31 (47%)	59 (52%)
Rural	20 (42%)	35 (53%)	54 (48%)
Chemotherapy administered?			
Yes	38 (79%)	49 (74%)	87 (76%)
No/Unknown	10 (21%)	17 (26%)	27 (24%)
Radiotherapy administered?			
Yes	13 (27%)	16 (24%)	29 (25%)
No	35 (73%)	50 (76%)	85 (75%)
Patient received surgery?			
Yes	23 (48%)	28 (42%)	51 (45%)
No/Unknown/Not applicable	25 (52%)	38 (58%)	63 (55%)

**TABLE 3:** Year‐by‐year breakdown of pediatric cancer diagnoses at study institution: Yearly amount of pediatric cancer diagnoses at our institution between 2018 and 2022.

Year	Number of diagnoses
2018	25
2019	24
2020	28
2021	25
2022	30

**TABLE 4:** Year‐by‐year population and percent increase of Eastern North Carolina: yearly population and percent increase of Eastern North Carolina between 2018 and 2022 per office of budget and management state demographer.

Year^a^	Population	Percent increase from year prior
2018	2 803 407	N/A
2019	2 818 907	0.55%
2020	2 831 495	0.44%
2021	2 859 575	0.98%
2022	2 890 516	1.10%

^a^ Data available at: https://demography.osbm.nc.gov/explore/dataset/county‐population‐totals/export/?flg=en‐us&disjunctive.vintage&disjunctive.estimateprojection&disjunctive.msa&disjunctive.cog&disjunctive.region&disjunctive.county&sort=region.

**TABLE 5:** Radiation therapy technique and site treated: radiation technique and goal‐of‐therapy for radiation therapy patients broken down by pre‐ and post‐COVID diagnoses.

	Pre‐COVID onset (%) (*n* = 9)	Post‐COVID onset (%) (*n* = 13)	Total (%) (*n* = 22)
Radiation technique			
3D CRT	4 (44%)	2 (15%)	6 (27%)
IMRT	5 (56%)	11 (85%)	16 (73%)
Site treated			
CNS	3 (33%)	7 (50%)	10 (43%)
WLI	1 (11%)	1 (7%)	2 (9%)
CSI	0 (0%)	1 (7%)	1 (4%)
Abdomen	3 (33%)	2 (14%)	5 (22%)
Trunk/Extremity	2 (22%)	3 (21%)	5 (22%)

*Note:* Column totals for the post‐COVID and Total groups are 14 and 23 as one patient received both lung and abdominal RT.

Abbreviations: 3D CRT, Three‐dimensional conformal radiation therapy; CNS, central nervous system; CSI, craniospinal irradiation; IMRT, intensity‐modulated radiation therapy; WLI, whole lung irradiation.

Analysis for differences between the pre‐ and post‐COVID groups stratified by race and age at diagnosis via Fisher's exact test did not reveal any significant variation (LR = 4.1 and 26.0, respectively; *p* = 0.667 and 0.099, respectively). Analysis based on rural versus urban patient home county was not significant (*p* = 0.231). Cancer primary site was not different between groups (*p* = 0.510). Finally, average distance traveled was not different between groups (*p* = 0.592). No difference was observed pre‐ and post‐COVID onset for the incidence of CNS or hematologic cancers (*p* = 0.379 and 0.674, respectively).

### 3.2 Overall Treatment Analysis

Information on the breakdown in treatment distribution for chemotherapy, RT, and surgery can be found in Table 1. No difference in the number of cancer diagnoses was evident for the number of patients in each cohort that received any single modality in the pre‐ and post‐COVID onset groups (*p* = 0.541, 0.731, and 0.560 for chemotherapy, RT, and surgery, respectively). Regarding surgery, 13 of the 23 patients (57%) that underwent surgery for a pediatric cancer diagnosis in the pre‐COVID onset group had surgery prior to any other treatment, compared with 20 of the 28 (71%) in the post‐COVID onset group. No difference was seen among the proportion of patients that received upfront versus post‐initial therapy surgery in the pre‐ and post‐COVID onset groups (*p* = 0.580).

### 3.3 Radiotherapy Analysis

**FIGURE 4:**
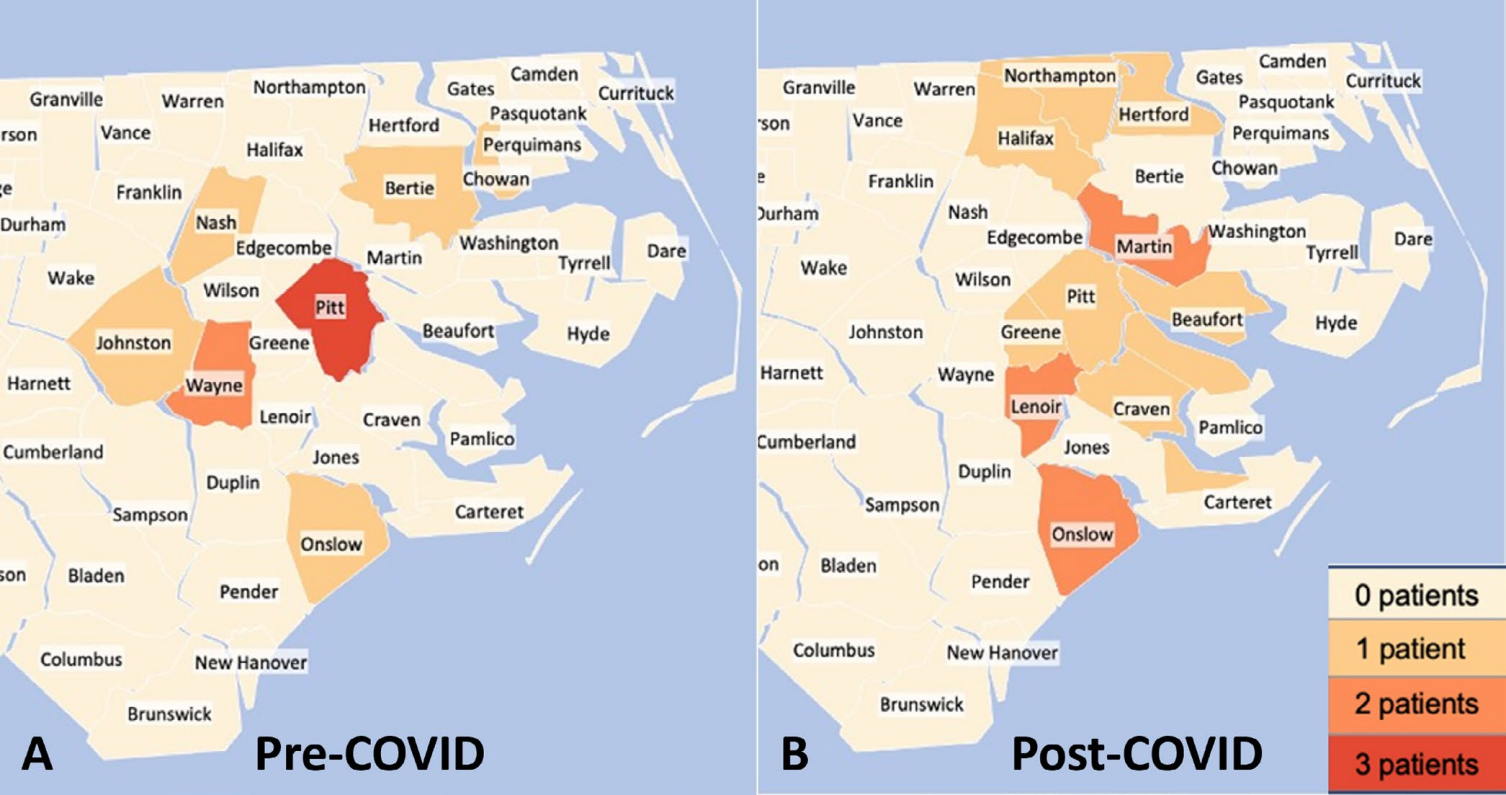
Heat maps of pediatric patients diagnosed with cancer and treated with radiotherapy at our institution pre‐ and post‐COVID onset (Panels A and B, respectively). Increasingly dark red corresponds to increasing frequency of patients diagnosed with cancer coming from the labelled county. Pre‐COVID group had nine patients, post‐COVID group had 13 patients.

Of the 29 patients that received RT, 22 had the entirety of their RT at our institution. For this subset of patients, no difference was observed pre‐ and post‐COVID onset for either the racial composition or age of patients (LR = 2.0 and 14.9, respectively; *p* = 0.371 and 0.249, respectively). However, the post‐COVID onset group receiving RT did see 4.5 times more patients coming from rural counties than the pre‐COVID group (*p* = 0.027); a heatmap depicting patient home counties pre‐ and post‐COVID onset can be seen in Figure 4. Distance traveled by patients in this group pre‐ and post‐COVID onset was not statistically different (*p* = 0.420). Additionally, the post‐COVID onset group saw increased diagnoses of patients with CNS cancers compared with the pre‐COVID onset group (*p* = 0.013). Post‐COVID onset, more non‐hematologic cancers were treated with RT at our institution compared with pre‐COVID onset (*p* = 0.049). No difference was seen pre‐ and post‐COVID onset for patients receiving chemotherapy or surgery in addition to RT at our institution (*p* = 0.962 and 0.597, respectively).

Median number of fractions pre‐COVID and post‐COVID onset were 28 and 31 respectively; the average number of fractions pre‐ and post‐COVID were 23 and 30. There was no difference in the estimated cost patients incurred as a result of their RT (pre‐COVID onset average = $277 per patient, post‐COVID onset average = $466 per patient; *p* = 0.144). Finally, no difference was seen pre‐ and post‐COVID onset in the number of patients receiving RT with concurrent chemotherapy (*p* = 0.245).

The majority of patients received intensity modulated radiation therapy (Table 5). However, there was no significant association between radiation technique and pre‐ and post‐COVID diagnosis (*p* = 0.13). No patients in our cohort received proton therapy at any point during the study period. The majority of treated sites were CNS tumors, particularly in the post‐COVID period (Table 5). However, there was no significant association between the site of treatment and pre‐ and post‐COVID diagnosis (*p* = 0.71). Of note, craniospinal irradiation (CSI) was not included in the latter Chi‐Square analysis due to a low sample size of zero CSI cases in the pre‐COVID period and one case in the post‐COVID period.

## 4 Discussion

Disparities in social determinants of health were highlighted by the COVID‐19 pandemic, with a disproportionate impact on certain vulnerable populations including the pediatric oncology population [25]. The pandemic presented many challenges for pediatric cancer patients due to delays in access to care [26]. This delay was exacerbated for patients who already had reduced access to care prior to the pandemic, such as those residing in rural counties [27]. Our study is novel in that we assessed the impact of the COVID‐19 pandemic in a vulnerable and understudied patient population.

We observed an increase in cancer diagnoses among patients from rural counties compared with urban counties post‐COVID onset, though this trend did not meet statistical significance for the cohort as a whole. This trend was significant in patients receiving RT, with 4.5 times more patients traveling from rural counties post‐COVID onset (*p* = 0.027). We initially thought this finding may be reflective of a shift in care, as some hospitals and treatment centers across the nation were temporarily closed due to staffing difficulties or reduced patient volume during the pandemic. The occurrence of such a shift is a potentially important factor to consider as hospital systems respond to external disasters, such as pandemics and mass casualty events. Literature on this shift is limited, although some literature reports on the difference in urban versus rural responses in healthcare during COVID‐19 [28, 29]. One such example is the increase in access to cancer surgery in urban areas as compared to rural ones, which may be a multifactorial trend not unlike access to RT for pediatric cancer patients, parents, involving physicians, hospital systems, payers, and patients themselves making choices that ultimately result in increasing centralization of healthcare resources [30]. However, Martin General Hospital was the only hospital in eastern North Carolina to close during the pandemic, and it only did so in August of 2023, after the time of our study cohort [31]. Additionally, the literature reports the increased usage of telehealth in cancer care secondary to the COVID pandemic, which may be reflective of both the practical need to avoid face‐to‐face encounters to limit disease transmission but also to potentially alleviate the burden faced by an increasingly rural group of patients when it comes to travel. Another plausible explanation for the increase in rural RT patients post‐COVID may be increased migration of this population to rural areas during the pandemic. We noted this effect in our study, evidenced by the increased population spread observed in our heat maps in the post‐COVID period for both patients as a whole (Figure 3) and for those who received radiation (Figure 4). The USDA reported non‐metropolitan areas saw a 0.12% increase in population from mid‐2021 to mid‐2022, which contrasts with the 0.09% decline demonstrated in the previous year and the overall trend of a 0.08% decline between 2010 and 2017 [32]. Rural North Carolina as a whole saw a 2.7% increase in population between 2020 and 2023, which is nearly as much as the increase in the previous decade [33]. Eastern North Carolina in particular saw an increased influx of population in the post‐COVID time period of our study, with roughly double the annual population increase in 2021 and 2022 as the years prior, shown in Table 4.

However, we could not report a significant association concerning distance travelled in the pre‐ versus post‐COVID onset groups, for either our entire study population or for the sub‐analysis of patients receiving RT at our institution. We noted fewer total patients coming from counties farther from our institution relative to counties closer to our institution, which could explain this finding. Additionally, there may be an individualized threshold in which travel becomes a barrier to patients receiving treatment, though this could not be elucidated based on our analysis. Finally, the finding in which there was an increase in patients coming from rural counties compared with urban during the COVID pandemic despite there being no significant change between distance travelled and the onset of the COVID pandemic could be the result of the relatively large area a patient could live within a county despite it being considered rural. It is possible that enough patients lived just across the county line of multiple counties and thus were close enough for the actual distance travelled to be insignificant despite our institution receiving increasingly more rural patients as regional centers closed or patients were diverted. It is also possible that some patients living farther away were able to stay in temporary housing facilities near our institution, though one of these facilities was closed during the pandemic while others had various restrictions during this time, making it difficult for us to factor in these variables to our analysis.

Trends towards a higher cost for travel were evident in the post‐COVID period in patients receiving RT. Nonetheless, given our relatively small sample size, these trends did not meet statistical significance. These findings may be due to the relative scarcity of RT centers in our region, particularly those that treat pediatric patients. This scarcity is more important to assess in patients residing in rural communities as there is a clear imbalanced distribution of radiation oncology facilities in these regions [9]. Our institution serves a large catchment area with limited RT sites within the region. Patients may have had limited options and may have continued to come to our institution despite the COVID‐19 pandemic. If this is true for other institutions as well, the institutions at which patients sought treatment during this timeframe may not have changed. This fact, in conjunction with no observed change in the increase in oncologic diagnoses in pediatric patients during the COVID‐19 pandemic, would seem to indicate that the distance traveled and subsequent cost of travel remained consistent. However, it is worth noting that inflation rose to nearly 9% in 2022, up from two to 3% prior to 2020, prior to receding back down to around 3% in 2023 [34], which may have still had a detrimental effect on the ability for underserved patients to pay for healthcare‐related costs. The COVID‐19 pandemic is thought to have exacerbated already existent racial disparities in healthcare, particularly in the Black and Latinx communities [35]. To expand on this, we sought to investigate whether the pandemic increased racial disparities in the pediatric cancer population by analyzing the association between race and pre‐ versus post‐COVID diagnosis among radiotherapy patients; our study did not find this association to be significant.

Central nervous system (CNS) and hematologic cancer types were the most prevalent in our study population pre‐COVID onset, and both of these cancer types had an increase in cases seen at our institution post‐COVID onset. We did not find a significant association between the number of diagnosed CNS tumors pre‐ versus post‐COVID onset for the cohort as a whole, though for patients receiving RT, there was an increase in the number of patients diagnosed with a CNS cancer post‐COVID onset (*p* = 0.018); a trend toward increased diagnoses of CNS tumors post‐COVID‐19 may be a result of delay in presentation and/or treatment initiation rather than a true increase in incidence of disease. This potential delay in presentation may have been due to fears in seeking care or a decrease in the number of primary care provider appointments, urgent care visits, or ED visits, which have been supported in prior literature [36, 37]. We also found an increase in the number of patients treated with RT for non‐hematologic cancers in the post‐COVID onset group; this may have been driven in part by the increase in CNS cancer patients treated with RT and by the increase in the number of patients diagnosed and treated with RT post‐COVID onset. The more straightforward interpretation of these trends would be that COVID shifted cancer care to a more centralized model, with rural/regional centers shutting down due to inadequate staffing, funding, and patient census, thus increasing the number of rural patients coming to our institution for diagnosis and treatment. This would be primarily driven by the increased number of patients with CNS cancers in the post‐COVID group, which are more commonly treated with RT than patients with hematologic malignancies. Another potential interpretation, albeit more unlikely, is that the COVID pandemic increased the incidence of cancer among pediatric patients within the catchment area of our institution.

Our study was subject to a number of limitations, such as the retrospective nature of the study, the relatively small sample size, and our data being limited to one tertiary care center. Another potential limitation was not having specific information regarding if the patients/their families stayed at temporary housing near the hospital, which may not have necessitated a daily commute. However, one of our temporary housing facilities was closed during the pandemic while another had numerous restrictions during this time, limiting the impact of inclusion of these facilities on the results of our study. Due to our large rural catchment area, our institution may not be directly comparable to other tertiary care centers. Our results may also be limited by hidden transportation costs outside of mileage, including toll fees and car ownership and maintenance. Despite its limitations, our study provides insight into factors that affect access to care when considering the COVID‐19 pandemic.

## Author Contributions

M.P.: Writing – original draft, Writing – reviewing and editing, conceptualization, data curation; M.C.L.: Writing – original draft, writing – reviewing and editing, data curation, visualization, formal analysis; G.E.: Writing – reviewing and editing, data curation, methodology, formal analysis, visualization; M.G.: Writing – reviewing and editing, data curation; D.G.: Writing – reviewing and editing, data curation; C.C.: Writing – reviewing and editing, supervision, validation; A.W.J.: Writing – reviewing and editing, investigation, supervision; A.B.: Writing – reviewing and editing, investigation, supervision, project administration.

## Ethics Statement

This project was certified exempt by our institution's institutional review board: University and Medical Center IRB 22–002292 “Pediatric Oncology Outcomes Database”. This article does not contain any studies with human participants or animals performed by any of the authors.

## Consent

Consent for publication was covered under the certified exemption by our institutional review board.

## Conflicts of Interest

The authors declare no conflicts of interest.

## Data Availability

The data that support the findings of this study are available from the corresponding author upon reasonable request.
